# Hydrolysable tannin-based diet rich in gallotannins has a minimal impact on pig performance but significantly reduces salivary and bulbourethral gland size

**DOI:** 10.1017/S1751731116002597

**Published:** 2016-12-22

**Authors:** G. Bee, P. Silacci, S. Ampuero-Kragten, M. Čandek-Potokar, A. L. Wealleans, J. Litten-Brown, J.-P. Salminen, I. Mueller-Harvey

**Affiliations:** 1 Agroscope Posieux, Tiolyere 4, 1725 Posieux, Switzerland; 2 Agricultural Institute of Slovenia, Hacquetova ulica 17, 1000 Ljubljana, Slovenia; 3 Department of Agriculture, University of Reading, Reading RG6 6AR, UK; 4 Department of Chemistry, University of Turku, 20500 Turku, Finland

**Keywords:** pigs, growth performance, meat quality, boar taint, tannins

## Abstract

Tannins have long been considered ‘anti-nutritional’ factors in monogastric nutrition, shown to reduce feed intake and palatability. However, recent studies revealed that compared with condensed tannins, hydrolysable tannins (HT) appear to have far less impact on growth performance, but may be inhibitory to the total activity of caecal bacteria. This in turn could reduce microbial synthesis of skatole and indole in the hindgut of entire male pigs (EM). Thus, the objective of this study was to determine the impact of a group of dietary HT on growth performance, carcass traits and boar taint compounds of group housed EM. For the study, 36 Swiss Large White boars were assigned within litter to three treatment groups. Boars were offered *ad libitum* one of three finisher diets supplemented with 0 (C), 15 (T15) or 30 g/kg (T30) of HT from day 105 to 165 of age. Growth performance, carcass characteristics, boar taint compounds in the adipose tissue and cytochrome P450 (CYP) isoenzymes CYP2E1, CYP1A2 and CYP2A19 gene expression in the liver was assessed. Compared with C, feed efficiency but not daily gain and daily feed intake was lower (*P*<0.05) in T15 and T30 boars. Except for the percentage carcass weight loss during cooling, which tended (*P*<0.10) to be greater in T30 than C and T15, carcass characteristics were not affected by the diets. In line with the numerically lower androstenone level, bulbourethral and salivary glands of T30 boars were lighter (*P*<0.05) than of T15 with intermediate values for C. Indole level was lower (*P*<0.05) in the adipose tissue of T30 than C pigs with intermediate levels in T15. Skatole levels tended (*P*<0.10) to be lower in T30 and C than T15 pigs. Hepatic gene expression of CYP isoenzymes did not differ between-treatment groups, but was negatively correlated (*P*<0.05) with androstenone (CYP2E1 and CYP1A2), skatole (CYP2E1, CYP2A) and indole (CYP2A) level. In line with the numerically highest androstenone and skatole concentrations, boar taint odour but not flavour was detected by the panellists in loins from T15 compared with loins from C and T30 boars. These results provide evidence that HT affected metabolism of indolic compounds and androstenone and that they affected the development of accessory sex glands. However, the effects were too small to be detected by sensory evaluation.

## Implications

Despite the great growth efficiency of entire male pigs (EM), this production is hampered by the risk of malodorous compounds such as androstenone, skatole and indole in pork. Thus, dietary approaches among others are needed to minimise the incidence of tainted meat. Hydrolysable tannins (HT) can inhibit the activity of the microbial population in the hindgut, which is where a part of the malodourous compounds are generated. This study reveals that at 3% dietary inclusion level, HT reduced the overall level of the relevant compounds in pork but the reduction was not sufficient to be perceived by sensory panel members.

## Introduction

Traditional silvopastoral systems for the production of Iberian ham in south-western Spain and Portugal have often relied on acorn-based diets to produce high-quality pork products. These products have been believed as antiquity to have unique flavour such as improved aroma, tenderness and juiciness, as well as a distinctive ‘bitter’ aroma (Cava *et al*., 2000). Many of these changes are likely attributable to the fatty acid composition of woodland diets, but the presence of tannins in the acorns cannot be discounted.

Tannins have long been considered ‘anti-nutritional’ factors in monogastric nutrition, shown to reduce feed intake and palatability, growth rates and feed efficiencies (Mueller-Harvey, 2006). However, tannins form many structurally diverse groups and each structural change may alter their impact and interaction of the feed with the host animal; generalisations about the effects and function of dietary tannins must not be based upon studies with tannins from a single plant species, but should be drawn from studies that address the wide range of available tannin chemistry (Hagerman *et al*., 1992). Many of the available studies on pigs focussed solely on the impacts of condensed tannins, which are found in a few forage legumes, faba beans (Mariscal-Landín *et al*., 2002), lupins (Ferguson *et al*., 2003) and also sorghum grains (Cousins *et al*., 1981). However, woodland diets can also contain HT (Cantos *et al*., 2003) and these may have different potencies and modes of actions as compared with condensed tannins. Recent studies have shown that the HT from chestnut have far less impact on growth rates and feed intake in grower and finisher pigs (Antongiovanni *et al*., 2007; Stukelj *et al*., 2010).

Interestingly, *in vitro* incubation of swine caecal inoculum with different concentrations of HT linearly reduced total gas production and the levels of acetic, propionic and *n*-butyric acid, but not the acetic to propionic acid ratio, suggesting an inhibitory effect on the total activity of caecal bacteria (Biagia *et al*., 2010). These findings are of interest especially in the production of EMs where the synthesis of skatole and indole, two compounds responsible for boar taint, could, therefore, be reduced in the colon. Thus, the objectives of this study were to evaluate the potential of dietary HT supplementation to control boar taint-related compounds and to determine their impact on growth performance, carcass characteristics, meat and sensory traits of pork from EMs.

## Material and methods

### Animals and diets

The Swiss Cantonal Committee for Animal Care and Use approved all procedures involving animals.

The experiment included a total of 36 Swiss Large White EMs originating from 12 litters. From weaning until the start of the experimental period (mean±SE BW=50.4±1.11 kg; age=105±0.3 days), EMs were group-penned and had *ad libitum* access to a commercial starter (mean±SE BW=10.8±0.44 to 27.2±0.25 kg) and grower diet (mean±SE BW=27.2±0.25 to 50.4±1.11 kg). At the end of the starter period, the littermates were allocated to the three experimental diets according to BW. The three diets consisted of a negative control diet (C) with no added tannins and two diets containing per kg of diet 15 (T15) or 30 g (T30) of supplementary tannins, respectively (Table 1). All diets were formulated to be isonitrogenous and isocaloric and tannin supplement, which originated from chestnut (Silvateam, San Michele Mondovì, Italy), was interchanged by straw meal. The experimental diets were offered *ad libitum* in a pelleted form. The pigs were reared in the finisher period in group pens, equipped with an automatic feeder and individual pig recognition system (Schauer Maschinenfabrik GmbH & Co. KG, Prambachkirchen, Austria) as described previously by Bee *et al*. (2008), which allowed the determination of individual feed intake in group housed pigs.

**Table 1:** Feed ingredients (%), nutrient and tannin composition of the experimental diets

	Treatment^1^		
	Control	T15	T30
Wheat	69.4	69.4	69.4
Barley	10.0	10.0	10.0
Dried sugar beet pulp	6.80	6.80	6.80
Potato protein	6.20	6.20	6.20
Fat blend^2^	1.48	1.48	1.48
Straw meal	3.00	1.50	
Hydrolysable tannins^3^		1.50	3.00
Dicalcium phosphate	1.126	1.126	1.126
Calcium carbonate	0.820	0.820	0.820
NaCl	0.280	0.280	0.280
l-Lysine HCl	0.166	0.166	0.166
dl-Methionine	0.006	0.006	0.006
l-Threonine	0.022	0.022	0.022
Pellan^4^	0.300	0.300	0.300
Mineral–vitamin premix^5^	0.400	0.400	0.400
Analysed nutrient and tannin composition (g/100 kg DM)			
Total ash (g/kg)	45.8	44.4	43.8
Crude fibre (g/kg)	29.1	23.3	25.3
CP (g/kg)	164.6	162.3	161.3
Crude fat (g/kg)	32.9	33.2	34.9
Total hydrolysable tannin (g/kg)	Nd	7.099	14.805
Ellagitannin (g/kg)	Nd	0.834	1.505
Gallotannin (g/kg)	Nd	5.234	11.384
Gallic acid (g/kg)	Nd	1.030	1.915
Calculated DE content (MJ/kg DM)^6^	16.80	16.80	16.80

DM, dry matter; DE, digestible energy.

1 Control=standard finisher diet without addition of chestnut tannin powder; T15=standard finisher diet with addition of 1.5% of chestnut tannin powder; T30=standard finisher diet with addition of 3% chestnut tannin powder.

2 50% fat from beef and 50% fat from swine.

3 Chestnut powder.

4 Binder that aids in pellet formation.

5 Supplied the following nutrients per kg of diet: 20 000 IU vitamin A, 200 IU vitamin D_3_, 39 IU vitamin E, 2.9 mg riboflavin, 2.4 mg vitamin B_6_, 0.010 mg vitamin B_12_, 0.2 mg vitamin K_3_, 10 mg pantothenic acid, 1.4 mg niacin, 0.48 mg folic acid, 199 g choline, 0.052 mg biotin, 52 mg Fe as FeSO_4_, 0.16 mg I as Ca(IO)_3_, 0.15 mg Se as Na_2_Se, 5.5 mg Cu as CuSO_4_, 81 mg Zn as ZnO_2_, 15 mg Mn as MnO_2_.

6 The DE coefficients from each feed ingredient were obtained from the Swiss (Agroscope, 2015) database, respectively. Taking into account the relative amount of each feed ingredient in the diet, DE content was calculated.

### Slaughter procedure and carcass measurements

One week after pigs reached 100 kg BW, they were selected for slaughter. Feed was withdrawn 16 h before slaughter by negating access to the feeder for the selected pigs. Avoiding all unnecessary stress, the EMs were walked ~100 m to the stunning area, and allowed to rest for 10 min before they were subjected to CO_2_ stunning for 100 s, after which they were exsanguinated, scalded, mechanically dehaired and eviscerated. At this point the internal organs were removed, and the liver, kidney, testicles, salivary (mandibular) and bulbourethral glands were weighed. Within 2 min after evisceration, liver excisions were rinsed with a phosphate-buffered saline solution and stored at −20°C in RNA-Later solution (1018087; Qiagen, Basel, Switzerland) until RNA extraction. The hot carcasses were then weighed and the pH and temperature of the *longissimus dorsi* muscle (LD) was measured at the 10^th^ rib location before being refrigerated at 3°C for 1 day. One day *postmortem*, the left cold carcass sides were weighed and dissected into the major primal cuts (shoulder, loin, ham and belly) and trimmed free of all external fat as previously described (Bee *et al*., 2004).

### Feed analysis

Feed samples were milled using a 1.0-mm sieve (Brabender, Duisburg, Germany) before analysis. Dry matter content was determined after samples were dried at 105°C for 3 h and ash content was subsequently determined after incineration at 550°C. Nitrogen was determined using Kjeldahl procedure (Leco FP-2000 analyser; Leco, Mönchengladbach, Germany) and CP calculated by 6.25×N. Crude fibre was analysed after digestion with successively H_2_SO_4_ and KOH, washed with acetone, dried at 130°C and finally ashed (EN 71/393, ISO 6865:2000, VDLUFA 6.1.4). Crude fat of diets was determined as petrol ether extract after an acidic hydrolysis (ISO 6492:1999, VDLUFA 5.1.1).

The content of gallic acid and HT in the diets were determined as recently described by Johnson *et al*. (2014). In brief, phenolic composition of the chestnut tannin extract and the four diet samples were quantified using 20 mg of finely ground powder extracted in 2×1.4 ml of acetone : water (8 : 2, v/v) for 3 h with a Vortex-Genie 2T mixer (Scientific Industries, Bohemia, NY, USA). After 10 min of centrifugation at 16 000×**g** (Eppendorf centrifuge 5402; Eppendorf AG, Hamburg, Germany), supernatant was stored in a separate vial and the extraction was repeated on the original tissue with fresh solvent. Acetone was removed from the pooled extracts using an Eppendorf concentrator 5301 (Eppendorf AG) and the sample was freeze-dried. Before analysis, sample was dissolved in 2 ml of water and filtered through a 0.20-µm polytetrafluoroethylene filter. Phenolics were analysed from each sample using ultra performance liquid chromatography with diode array and electrospray MS detectors (UPLC-DAD-MS, Waters Acquity UPLC and Waters Xevo TQ triple quadrupole mass spectrometer; Waters Corporation, Milford, MA, USA). We used a Waters Acquity UPLC BEH Phenyl (1.7 ml, 2.1 9100 mm) column with CH_3_CN (A) and 0.1% HCOOH as eluents (Engström *et al*., 2015). Individual HTs were identified on the basis of their UV and MS spectra (Moilanen *et al*., 2013). Gallic acid was quantified in gallic acid equivalents, all gallic acid derivatives in pentagalloylglucose equivalents and ellagitannins as tellimagrandin I equivalents.

### Analysis of indole, skatole and androstenone concentrations in the adipose tissue

Androstenone, skatole and indole concentrations in the adipose tissue were analysed according to Ampuero Kragten *et al*. (2011) and based on the method previously described by Hansen-Møller (1994). In brief, the adipose tissue samples were liquefied in a microwave oven for 2×2 min at 250 W. The liquefied lipids were centrifuged for 2 min at room temperature. The water was then removed and 0.5 ml of water-free liquid fat, kept at around 47°C, was placed in 2.5-ml Eppendorf tubes in duplicates and an internal standard was added (1 ml methanol containing 0.496 mg/l androstanone and 0.050 mg/l 2-methylindole). After vortexing for 30 s, the tubes were incubated for 5 min at 30°C in an ultrasonic water bath, kept at 0°C in ice-water bath for 20 min and then centrifuged at 11 000×**g** for 20 min. Finally, the liquid fraction was filtered (0.2-μm filter) and transferred into a vial for androstenone, skatole and indole analysis with an HPLC system. Concentrations were expressed per gram of adipose tissue. The quantification limits were 0.3 μg/g adipose tissue for androstenone and 0.03 μg/g adipose tissue for skatole and indole.

### RNA isolation

Total RNA was extracted using a Nucleospin RNA XS kit (740902; Macherey-Nagel, Oensingen, Switzerland) following the procedure provided by the manufacturer. In brief, after thawing, 3 to 4 mg of liver tissue was homogenised in a MiniLys Bertin (Labgene, Chatel-St-Denis, Switzerland) using CK14 Precellys Lysing Kit (KT03961-1-203; Labgene) in 0.3 ml of RA1 buffer of the Nucleospin RNA XS kit. The RNA concentration was determined using a NanoDrop ND 1000 spectrophotometer (Witec AG, Littau, Switzerland). The mean 260/280 absorbance ratio of the samples was 2.13.

### Primer design, reverse transcription and quantitative real-time PCR

For reverse transcription with the Quantitect Reverse Transcription kit (205311; Qiagen, Basel, Switzerland), which also includes a DNase I digestion step, 250 ng of RNA were used. The resulting reaction product was diluted 1 : 1 with RNAse-free water before further analysis. Primers for cytochrome P450 (CYP) isoenzyme *CYP1A2* and *CYP2E1* and *CYP2A19* were designed using Primer-Blast service (Ye *et al.*, 2012) offered by the National Institute of Health and verified for specificity using National Center for Biotechnology Information database (www.ncbi.nlm.nih.gov). Primers for the *CYP1A2* gene were the same as used by Rasmussen *et al.* (2011). The target and housekeeping gene primers and National Center for Biotechnology Information accession numbers are shown in Supplementary Table S1. For each primers pairs, the efficiency of amplification was determined in three independent experiments.

Genes encoding *CYP1A2*, *CYP2E1* and *CYP2A19* were evaluated for their expression in pig livers via quantitative real-time PCR, using a KAPA Sybr Fast qPCR Kit (KK 4602m; Kapa Biosystems, Labgene, Chatel-St-Denis, Switzerland) with an Eco Illumina-Real Time PCR System (Labgene). The programme consisted of a pre-incubation step at 95°C for 5 min, followed by 40 cycles of 5 s at 95°C and 20 s at 62°C. The expression of each targeted gene was evaluated using 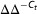 method (with efficiency corrections) and normalised using glyceraldehyde-3-phosphate dehydrogenase (GAPDH) as housekeeping genes. All the calculations were performed using Eco-Illumina Study software (Labgene).

### Sensory analysis

The sensory test aimed to determine differences in intensity of boar taint odour and flavour as well as additional sensorial characteristics of cooked loin chops related to the dietary HT supply. The panel, which consisted of eight judges (five females and three males, from 35 to 62 years of age), was previously trained to determine boar taint as well as juiciness and tenderness. In addition, by using LD samples collected from the experimental animals, judges completed four-training sessions in order to agree on the following three discriminant descriptors: ‘bitter’, ‘gummy’ and ‘astringent’. For the traits juiciness, tenderness, bitter, gummy and astringent a monadic linear scale ranging from 0 to 10 (0=low intensity, weak; 10=great intensity, strong) was used. By contrast, for the boar taint assessment the judges indicated the presence or absence of boar taint odour and flavour using R-index technique (O’Mahony, 1988). The judges could give one of the four following answers regarding boar taint odour and flavour in the test as compared with the control sample: S (the flavour and odour was definitely present), S? (flavour and odour was present but they were not sure), N? (flavour and odour was not present but they were not sure) or N (flavour and odour was not present and they were sure). Samples were offered in a randomised order to ensure that judges did not evaluate the same sample at the same time. The judges were instructed to begin with the first sample on the left side of the plate and follow it clockwise. They were also asked to assess the traits according to a defined sequence. First, they had to smell the meat immediately after removing the glass top and to evaluate boar odour intensity. They were then requested to chew and taste the meat sample and evaluate boar flavour, juiciness, tenderness, bitterness and gumminess intensities and astringent properties. Before starting the sensory evaluation of the following sample, they were asked to eat bread and rinse their mouth with light black tea.

The loin samples from C, T15 and T30 pigs originating from the same litter were served on the same dish, with an extra C sample as a control sample for the R-index test. The litters were randomised between the sessions. In four sessions (*n*=12, two sessions per week), the LD-strips originating from four EM were offered with a 10-min interval between servings. Data were recorded with the help of the Fizz Network data acquisition system and software (Version 2.0; Biosystems, Couternon, France).

### Statistical analysis

Data for growth performance, carcass characteristics and meat quality traits were analysed with the MIXED procedure of SAS. The model used included litter and experimental groups as fixed effects. The individual pig was the experimental unit for analysis of all data. Least-squares means were calculated and considered statistically significant at *P*⩽0.05 and tendencies were denoted at *P*⩽0.10 and >0.05. Pearson’s correlations between boar taint compounds and weight of testes, accessory sex glands and CYP gene isoform expression were determined using CORR procedure.

Parametric modelling of the continuous traits of the sensory analysis was performed using MIXED procedure of SAS. As residual diagnostics in the parametric linear mixed models indicated strong deviations from normality in most cases, the R package ‘robustlmm’ (Koller, 2013) was used to estimate the model parameters by a robustified REML algorithm (Koller, 2014). Inference was based on *t*-values (normal approximation), non-significant (*P*>0.05) interactions and (near) zero-valued variance components were step-wise excluded. The full model is given in the following equation:1
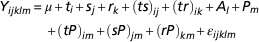



*μ* is the general mean; *t*_*i*_ the fixed effect of treatment *i*; *s*_*j*_ the fixed effect of session *j*; *r*_*k*_ the fixed effect of service *k*; (*ts*)_*ij*_ the interaction of treatment *i* with session *j*; (*tr*)_*ik*_ the interaction of treatment *i* with service *k*; *A*_ð_ the random effect of animal *l*; *P*_*m*_ the random effect of panellist *m*; (*tP*)_*im*_ the interaction of treatment *i* with panellist *m*; (*sP*)_*jm*_ the interaction of session *j* with panellist *m*; (*rP*)_*km*_ the interaction of service *k* with panellist *m*; *ε*_*iklm*_ the residual error.

The R-indices for boar odour and flavour were computed in Excel^®^ according to O’Mahony (1992) on the basis of Thurstonian modelling and signal detection theory of the categorical response values *v*. treatments. Inference concerning the R-indices was based on tables of critical values as published by Bi and O’Mahony (2007).

## Results

### Characterisation of hydrolysable tannin of chestnut

Per kilogram of the chestnut tannin extract consisted of 88.7 g ellagitannins, 450.9 g gallotannins and 37.5 g gallic acid. Gallotannins consisted mainly of nine different types of galloylglucoses having six to 14 galloyl groups, but also smaller levels of di-, tri-, tetra- and pentagalloylglucoses were found. The relatively low levels of ellagitannins consisted mainly of C-glycosidic ellagitannins such as vescalagin, castalagin and castavaloninic acid. In addition, the product contained small amounts of proanthocyanidins (syn. condensed tannins, 3.16 g/kg), consisting of prodelphinidin (48%) and procyanidin (52%) subunits. When included in the pelleted diets, the tannin levels amounted to 0.07% and 0.13% for ellagitannins and 0.46% and 1.01% for gallotannins in the T15 and T30 diets, respectively (Table 1). In addition, the percentage gallic acid amounted to 0.09% and 0.17% in the T15 and T30 diets, respectively. Diet C did not contain any detectable amounts of HT or gallic acid. In all the three experimental diets no condensed tannins were detectable.

### Growth performance, morphometric measurements and carcass characteristics

Tannin supplementation had no effect on average daily gain and average daily feed intake (Table 2). Nevertheless, gain-to-feed ratio was lower (*P*<0.05) in the tannin supplemented compared with the unsupplemented group mainly because of a combination of numerically slower growth and a numerically greater average daily feed intake. Apart from percentage cold loss that tended (*P*<0.10) to be greater in the T30 compared with the C and T15 groups, hot carcass weight, killing out percentage and carcass characteristics such as percentage lean meat, primal cuts and backfat were not affected by the experimental treatments. Compared with the T15 group, the weight of the salivary and bulbourethral glands was depressed (*P*<0.05) and that of the kidney tended (*P*<0.10) to be lower in EM of the T30 group, with reductions of 20%, 18% and 8%, respectively. Except for the kidney, EM of the C group displayed intermediate values.

**Table 2:** Effect of increasing inclusion levels of hydrolysable tannins in the finisher diet on growth performance, carcass characteristics and organ weights of entire males

	Treatment^1^				
Items	Control	T15	T30	SE	*P*-value
BW (kg)					
At birth	1.59	1.60	1.60	0.09	0.99
At 100 days of age	50.18	52.75	49.84	1.94	0.30
At 160 days of age	111.36	113.30	109.41	3.44	0.58
Average weight gain (g/day)	987	978	962	31	0.78
Average daily feed intake (g/day)	2583	2705	2621	87	0.43
Gain to feed (g/kg)	382^b^	363^a^	367^a^	5	0.02
Carcass characteristics					
Hot carcass weight (kg)	86.62	88.79	84.79	3.41	0.46
Carcass yield (%)	79.13	79.69	78.81	0.42	0.16
Cold loss (%)^2^	2.64^xy^	2.62^x^	2.86^y^	0.08	0.07
Lean percentage (%)^3^	55.67	55.36	55.91	0.46	0.60
Loin (%)	24.80	24.91	24.96	0.30	0.83
Ham (%)	18.49	18.18	18.55	0.23	0.30
Shoulder (%)	12.38	12.28	12.40	0.14	0.76
Belly (%)	18.66	18.87	18.52	0.23	0.43
Backfat (%)^4^	13.42	13.51	13.19	0.40	0.77
10^th^ rib backfat thickness (mm)	19	19	18	1	0.74
Organ weight					
Liver (g)	1805	1764	1740	59	0.53
Kidney (g)	370^y^	375^y^	342^x^	19	0.09
Testes (g)	635	634	606	37	0.68
Mandibular gland (g)	86^ab^	96^b^	77^a^	5	0.02
Bulbourethral gland (g)	183^ab^	199^b^	163^a^	13	0.03

^a,b^Values within a row with different superscript letters differ significantly at *P*⩽0.05. ^x,y^Values within a row with different superscript letters tend to differ significantly at *P*⩽0.10.

1 Control=standard finisher diet without addition of chestnut tannin powder; T15=standard finisher diet with addition of 1.5% of chestnut tannin powder; T30=standard finisher diet with addition of 3% chestnut tannin powder.

2 Weight loss of the hot carcass during cooling at 2°C for 24 h.

3 Sum of denuded shoulder, back and ham weights as percentage of cold carcass weight.

4 Sum of external fat from the shoulder, back and ham expressed as percentage of cold carcass weight.

### Meat quality, boar taint compounds and cytochrome P450 isoenzyme gene expression

Thaw losses were greater (*P*<0.05) in LD chops from EM of the T30 compared with T15 group, whereas other meat quality traits were not affected by the tannin supplementation (Table 3). Skatole and indole tissue levels were lower (*P*<0.10) in T30 compared with T15 pigs (Table 3). Compared with EM of the C group, only indole but not skatole levels in the adipose tissue were lower (*P*<0.05) in EM of the T30 group. Although not reaching a tendency (*P*=0.16), androstenone levels were lowest in the T30, highest in the T15 and intermediate in the C group.

**Table 3:** Effect of increasing inclusion levels of hydrolysable tannins in the finisher diet of entire males on meat quality traits and boar taint compounds in the adipose tissue

	Treatment^1^				
Items	Control	T15	T30	SE	*P*-value
Muscle pH					
At 45 min	6.53	6.51	6.51	0.05	0.93
At 1 day *postmortem*	5.57	5.57	5.59	0.02	0.68
Muscle temperature (°C)					
At 45 min	38.9	39.1	39.0	0.2	0.56
At 1 day *postmortem*	3.0	3.1	3.2	0.1	0.50
Meat colour					
L*^2^	48.8	49.5	50.0	1.0	0.63
Water holding capacity (%)					
Drip loss (48 h)	2.72	2.60	2.99	0.42	0.66
Thaw loss	7.65^xy^	6.64^x^	7.75^y^	0.37	0.07
Cooking loss	23.48	22.75	23.60	0.78	0.59
Shear force (kg)	6.72	6.26	6.43	0.34	0.49
Bovar taint compounds (μg/g adipose tissue)					
Androstenone	0.65	0.81	0.45	0.16	0.14
Skatole	0.19^x^	0.31^y^	0.19^x^	0.05	0.09
Indole	0.06^b^	0.05^ab^	0.03^a^	0.01	0.04

^a,b^Values within a row with different superscript letters differ significantly at *P*⩽0.05. ^x,y^Values within a row with different superscript letters tend to differ significantly at *P*⩽0.10.

1 Control=standard finisher diet without addition of chestnut tannin powder; T15=standard finisher diet with addition of 1.5% of chestnut tannin powder; T30=standard finisher diet with addition of 3% chestnut tannin powder.

2 L*=lightness (lower values=darker colour; greater values=lighter colour).

The messenger RNA CYP isoenzyme expression in the liver was not affected by the dietary treatments (Table 4). However, expression level of the *CYP1A2* and *CYP2A19* isoforms were numerically greater in the T30 compared with the T15 treatment.

**Table 4:** Effect of increasing inclusion levels of hydrolysable tannins in the finisher diet of entire males on hepatic messenger RNA (mRNA) cytochrome P450 (CYP) isoenzyme expression^1^

	Treatment^2^				
Items	Control	T15	T30	SE	*P*-value
*CYP1A2*	0.65	0.43	0.92	0.19	0.21
*CYP2A19*	0.59	0.47	0.94	0.29	0.15
*CYP1E2*	1.19	1.10	1.51	0.24	0.41

1 The mRNA expression of the CYP isoenzymes *CYP1A2*, *CYP2A19* and *CYP1E2* are normalised against the mRNA expression of glyceraldehyde-3-phosphate dehydrogenase.

2 Control=standard finisher diet without addition of chestnut tannin powder; T15=standard finisher diet with addition of 1.5% of chestnut tannin powder; T30=standard finisher diet with addition of 3% chestnut tannin powder.

The adipose tissue concentration of androstenone, skatole and indole was positively (*P*<0.05) correlated with the testes, mandibular gland and accessory sex gland weights (Table 5). Furthermore, gene expression of *CYP2E1* and *CYP1A2*, *CYP2E1* and *CYP2A*, and *CYP2A* decreased (*P*<0.05) with increasing tissue concentrations of androstenone, skatole and indole, respectively.

**Table 5:** Correlation coefficients between boar taint compound level in the adipose tissue and weight of testes, bulbourethral and salivary gland as well as on hepatic messenger RNA (mRNA) cytochrome P450 (CYP) isoenzyme expression^1^

	Androstenone	Skatole	Indole
Weight of			
Testes	0.581**	0.608**	0.409*
Bulbourethral gland	0.653**	0.565**	0.384*
Mandibular gland	0.632**	0.385**	0.341*
Relative expression			
*CYP2E1*	−0.383*	−0.381*	
*CYP2A19*		−0.533**	−0.429*
*CYP1A2*	−0.350*		

1 The mRNA expression of the CYP isoenzymes *CYP1A2*, *CYP2A19* and *CYP1E2* are normalised against the mRNA expression of glyceraldehyde-3-phosphate dehydrogenase.

*P<0.05, **P<0.01.

### Sensory analysis

Intensity of bitterness tended (*P*<0.10) to be lower and that of gumminess tended to be greater in the meat of C compared with the T15 and T30 pigs, respectively (Table 6). Presence or absence of boar taint odour and flavour of pork chops from EM was analysed by the R-index procedure (Table 7). In this method, the odour and flavour scores for samples from EM of the T15 and T30 group were compared with those of the C treatment. The R-indices represent the probability of detecting a difference in the presence or absence of boar odour and flavour between EM of a particular treatment and samples of EM of the C group.

**Table 6:** Effect of increasing inclusion levels of hydrolysable tannins in the finisher diet on sensory scores of loin chops from entire males^1^

	Treatment^2^				
Descriptors	Control	T15	T30	SE	*P*-value
Bitter	1.36^x^	1.68^y^	1.52^xy^	0.56	<0.10
Gummy	2.95^y^	2.50^xy^	2.42^x^	0.79	<0.10
Astringent	1.63	1.65	1.41	0.39	>0.10
Juiciness	2.54	2.87	2.82	0.62	>0.10
Tenderness	2.58	2.05	3.36	0.84	>0.10

^x,y^Values within a row with different superscript letters tend to differ significantly at *P*⩽0.10.

1 Scores are defined as 0=no intensity, weak; 10=very high intensity, strong.

2 Control=standard finisher diet without addition of chestnut tannin powder; T15=standard finisher diet with addition of 1.5% of chestnut tannin powder; T30=standard finisher diet with addition of 3% chestnut tannin powder.

**Table 7:** Probabilities of detecting the differences in boar taint odour and boar taint flavour by judges (R-index values (%)) between meat from entire male pigs fed the T15 or T30 diet compared with meat from entire male pigs fed the control diet^1^,^2^

Sensory attributes	T15	T30
Odour	59.00*	55.27
Flavour	55.37	43.26

*Judges were sure about the presence of boar odour in meat from T15 entire males compared with that of entire males of the control group.

1 Control=standard finisher diet without addition of chestnut tannin powder; T15=standard finisher diet with addition of 1.5% of chestnut tannin powder; T30=standard finisher diet with addition of 3% chestnut tannin powder.

2 The R-indices represent the probability of detecting a difference in the presence or absence of boar odour and flavour between samples of T15 and T30 entire males and samples of C entire males. An R-index of 50% or lower indicates no difference in terms of boar taint odour or flavour between meat from entire males fed the T15 or T30 diets and meat from entire males fed the control diet; a R-index above 50%+the critical value for *α* =0.05, indicates that judges were sure about the presence of boar odour or flavour in meat from T15 and T30 entire males compared with that of entire males of the control group. The critical value for the R-index was determined to be 7.21% (Bi and O’Mahony, 2007).

The R-index measures the difference between a sample (designated as the ‘signal’ sample) and a control sample (designated as the ‘noise’ sample) during a paired comparison of both. An R-index value of 100% indicates that samples are perfectly distinguishable, whereas a value of 50% indicates that both samples are equal and therefore the null hypothesis cannot be disregarded. A given confidence interval, together with the number of DF, will determine a critical value (R-index – 50%) above which meat from EM fed the HT-supplemented diets present boar taint, either in odour or in flavour, significantly greater than the meat from EM fed diet C. The critical value for the R-index was determined to be 7.21% (Bi and O’Mahony, 2007). Therefore, meat from T15 pigs displayed more (*P*<0.05) boar odour, but not boar flavour, compared with meat from C pigs. By contrast, meat from T30 and C pigs did not differ regarding boar odour and flavour.

## Discussion

Results of *in vitro* studies from Biagia *et al*. (2010) showed an inhibitory effect of tannins on the total activity of caecal bacteria. The large intestine and colon is the production site of two compounds, skatole and indole. Skatole and androstenone are the main compounds responsible for the incidence of boar taint and indole makes a minor contribution (Xue and Dial, 1997; Wesoly and Weiler, 2012). In several European countries with a large pig sector, there is a clear incentive to switch from barrows to EM production (Bee *et al*., 2015). In light of these facts, it is relevant to evaluate whether tannins might lower or even inhibit the production of indole and skatole and thereby reduce the incidence of boar taint. The major finding of the present study is that compared with the control at the greatest HT inclusion level, the diet rich especially in gallotannins resulted in reduced indole and numerically lower androstenone tissue levels, but the reductions were not sufficient to be perceived by the sensory panel. Puzzling were the findings at 1.5% HT inclusion level as adipose tissue levels of skatole and numerically of androstenone were greatest and accordingly boar odour perception was highest.

### Dietary effects on growth performance, carcass characteristics and organ weights

Despite tannin supplementation having no effect on growth rate and feed intake, gain-to-feed ratio was lower in both, the T15 and T30 treatment, compared with the C treatment. However, the lower feed efficiency had no impact on lean tissue and adipose tissue deposition. Recently, Čandek-Potokar *et al*. (2015) observed lower feed intake and only numerically lower growth rate and feed efficiency when EM were offered a diet supplemented with three compared with 2%, 1% or 0% chestnut tannin extract. By contrast, Antongiovanni *et al*. (2007) reported no differences in feed intake and feed efficiency of finisher pigs fed chestnut tannins at a 0.20% to 0.50% level. These results suggest that there is a dose-dependent effect of gallotannin-rich feed on growth performance.

The lower weights of the mandibular and bulbourethral glands and the numerically lower testes weight of T30 compared with T15 and C pigs partly corroborate the results of Čandek-Potokar *et al*. (2015). In line with its function as a pheromone-secreting gland, the weights of the mandibular glands were positively correlated with androstenone tissue levels. Similar in magnitude, the weights of testes and bulbourethral glands were also positively correlated with androstenone tissue levels. As reviewed by Dias *et al*. (2014), plant extracts rich in tannins and flavonoids can reduce LH and testosterone serum concentrations in male albino rats treated with increasing levels of these extracts over a 60-day period. Similarly, Abarikwu *et al*. (2014) reported that gallic acid directly suppressed steroidogenesis in male Wistar rats by decreasing androgenic enzyme activities. Thus, lower levels of gonadotrophins might decrease the androgenic activity of the testes and in turn understimulate the accessory sex glands, causing their regressive changes. This leads to the hypothesis that were tannins to be introduced into the diets of growing boars at a younger age, before the onset of puberty and the production of sex-related chemicals like androstenone, it is possible that the disruption of the hormone-controlled production of androgenic compounds might act more efficiently, reducing the levels of androstenone in slaughter weight boars.

### Dietary effects on skatole, indole and androstenone levels in adipose tissue

One of the main objectives of the present study was to assess the potential of HT to reduce the deposition of skatole and indole in the adipose tissue by affecting the microbial synthesis of these substances in the hindgut. This objective was based on promising results of *in vitro* and *in vivo* experiments of Biagia *et al*. (2010) suggesting that dietary HT trigger a shift from a predominantly proteolytic to a predominantly saccharolytic microbial metabolism. In the present study, we observed that with increasing dietary HT inclusion, indole concentration in the adipose tissue linearly decreased. The fact that sensory threshold for indole is 53 times lower than for skatole (Nagata and Takeuchi, 2003) it is not plausible that the observed decline in indole level was directly associated with the sensory assessment. Surprisingly, skatole tissue levels followed a curvilinear response pattern, as at 1.5% compared with 0% and 3% inclusion levels, skatole tissue concentrations were almost twice as high and way above the acceptance threshold (Meier-Dinkel *et al*., 2015). These findings partly corroborate those of Čandek-Potokar *et al*. (2015) who reported also a curvilinear relationship between dietary levels of chestnut tannin extract and the adipose tissue concentration of skatole. At a dietary HT concentration of 3%, 2% and 0% compared with 1%, skatole adipose tissue levels were two-, two- and three-times lower, respectively, and did not follow the concentrations determined in the colon (Čandek-Potokar *et al*., 2015). The lack of synchrony between skatole and indole content in the colon and their respective tissue levels is not surprising as absorption and hepatic clearance are, in addition to microbial synthesis, determinant factors for their final tissue concentrations (Wesoly and Weiler, 2012). Knarreborg *et al*. (2002) concluded from the strong correlation between the concentrations of indolic compounds in the tissue and blood within animals that a proportion of the indolic compounds produced was also absorbed. However, the weak relationships between animals emphasise the importance that individual hepatic clearance rate has on the level of these adipose tissue compounds. In accordance with Čandek-Potokar *et al*. (2015), the significance of liver metabolism is underlined in the present study by the negative relationship between the skatole and indole concentrations in the adipose tissue and the hepatic gene expression of *CYP2E1* and *CYP2A19*. These findings extend those of Čandek-Potokar *et al*. (2015) suggesting a link between dietary HT intake and gene expression and enzyme activity of two major enzymes of the phase 1 metabolism (Wiercinska *et al*., 2012). However, based on earlier results not the high molecular weight polyphenols, but their metabolites like the urolithins are responsible for these effects. Espín *et al*. (2007) showed that ellagic acid released from acorn ellagitannins in the jejunum are metabolised by the intestinal flora to yield urolithin metabolites, which are then absorbed into the bloodstream, conjugated in the liver and either excreted in the urine or undergo active enterohepaticcirculation. What the authors also showed that these metabolites are not stored in the organs like liver, lung, heart, kidney, muscle and adipose tissue. However, as reviewed by Korobkova (2015) these metabolites have the potential to alter CYP activity either via direct interactions with the enzymes or by affecting the CYP gene expression. The latter could be one possible mechanism linking the 3% HT intake, adipose tissue skatole and indole concentrations and hepatic gene expression of *CYP2E1* and *CYP2A19*. The reason for the unexpected results in the T15 group remains unclear. Nevertheless, the high skatole level in the T15 group concur with the numerically highest androstenone concentration and numerically lowest *CYP2E1* and *CYP2A19*. It has been shown in isolated pig hepatocytes that excessive concentrations of androstenone prevent *CYP2E1* induction by its substrate skatole, resulting in lower hepatic skatole clearance and therefore greater accumulation in the adipose tissue (Doran *et al*., 2002).

### Dietary effects on meat quality traits and sensory quality

Apart from small differences in thaw losses, being in tendency 1% unit lower in T15 compared with C and T30, meat quality was not affected by the dietary HT supplementation. The lack of HT impact on meat quality is comparable with other dietary approaches that seek to control boar taint with for instance inulin or raw potato starch (Pauly *et al*., 2008; Aluwe *et al.*, 2013). In line with the higher skatole and androstenone concentrations, members of the sensory panel could detect a stronger boar taint odour but not flavour in cooked loin chops of the T15 compared with the C and T30 groups. The finding that the sensitivity towards odour is stronger than towards flavour is in agreement with a recent study (Bonneau and Chevillon, 2012). As previously mentioned, skatole levels at T15 were above tolerable thresholds, whereas androstenone levels were rather low (Bonneau and Chevillon, 2012). This led us to conclude that the adverse reaction of the panellists towards loin from T15 pigs was primarily the result of the greater skatole and not the androstenone concentration. During the training sessions with loin chops from the experimental animals, the members of the panel agreed on the sensory descriptors bitterness, gumminess and astringency. Only two of the three descriptors tended to differ between dietary treatments. Although at very low-score levels, bitterness and gumminess scores tended to be greatest in the T15 and C groups, respectively. Surprisingly, the extent of astringency, which is often associated with tannins did not differ between-treatment groups. All in all, apart from boar taint, the sensory analysis revealed no noticeable concern regarding sensorial properties of loins from pigs fed HT-supplemented diets.

## Conclusion

The results of this study confirm that up to 3% HT in a finisher diet of EM has only a marginal impact on growth performance, carcass characteristics and meat quality. It appears that a HT-based diet rich in gallotannins may to some extent be effective at tackling boar taint, but only at the greatest inclusion level. The results obtained at the lower inclusion level are puzzling because they suggest that HT affected either indolic compound production and/or absorption positively in the hindgut or hampered hepatic clearance by reducing CYP isoenzyme expression through unknown mechanisms. Further studies are warranted to unravel the effect of HT on the gut microbiome and liver metabolism, which might lead to additional feeding strategies for EM and to strategies for dealing with boar tainted pork.

## Supplementary material

For supplementary material accompanying this paper visit http://dx.doi.org/10.1017/S1751731116002597. click here to view supplementary material
