# Supplementary material for: Hydrolysable tannin-based diet rich in gallotannins has a minimal impact on pig performance but significantly reduces salivary and bulbourethral gland size
Source: Animal. 2016 Dec 22;11(9):1617–25. doi: 10.1017/S1751731116002597 (PMC5561437; doi:10.1017/S1751731116002597)
Supplement: Supplementary file 1 [file S1751731116002597sup001.docx]

Hydrolysable tannin-based diet rich in gallotannins has a minimal impact on pig performance but significantly reduce salivary and bulbo-urethral gland size

G. Bee, P. Silacci, S. Ampuero-Kragten, M. Čandek-Potokar, A.L. Wealleans, J. Litten-Brown, J.-P. Salminen, I. Mueller-Harvey

Supplementary Table S1 Primer and probe sequences of cytochrome P450 isoenzymes (CYP)1A2, CYP2E1, CYP2A19 and glyceraldehyde 3 phosphate dehydrogenase (GAPDH)

| Gene | Accession Number | sequence (5’ to 3’) | Product length | Amplification efficiency % |
| --- | --- | --- | --- | --- |
| CYP1A2 | NM_001159614.1 | Forward primer: CTG CAA TTC CTG AGG AAA ATG G | 76 | 97 |
|  |  | Reverse primer: CGC TTG TGA TGT CCT GGA TAC A |  |  |
| CYP2E1 | NM_214421.1 | Forward primer: CTG GAG GCA CTC AGG AAG AC | 230 | 103 |
|  |  | Reverse primer: CTT CCA GGC AGG TAG CGT AG |  |  |
| CYP2A19 | NM_214417.1 | Forward primer: TGG ATG AGA ACG GGC AGT TT | 99 | 107 |
|  |  | Reverse primer: AGA GCT CCA TTC TAG CCA GAC CTT |  |  |
| GAPDH | NM_001206359.1 | Forward primer: GTC GGT TGT GGA TCT GAC CT | 210 | 100 |
|  |  | Reverse primer: AGC TTG ACG AAG TGG TCG TT |  |  |
